# Part II: Living Life: A Meta-Synthesis Exploring Recovery as Processual Experiences

**DOI:** 10.3390/ijerph18116115

**Published:** 2021-06-06

**Authors:** Mona Sommer, Stian Biong, Marit Borg, Bengt Karlsson, Trude Klevan, Ottar Ness, Linda Nesse, Jeppe Oute, Rolf Sundet, Hesook Suzie Kim

**Affiliations:** 1 Department of Health, Social and Welfare Studies, Faculty of Health and Social Sciences, University of South-Eastern Norway (USN), 3040 Drammen, Norway; stian.biong@usn.no (S.B.); marit.borg@usn.no (M.B.); bengt.karlsson@usn.no (B.K.); trude.goril.klevan@usn.no (T.K.); Jeppe.O.Hansen@usn.no (J.O.); rolf.sundet@usn.no (R.S.); hsuziekim@comcast.net (H.S.K.); 2 Department of Education and Lifelong Learning, Norwegian University of Science and Technology, 7042 Trondheim, Norway; Ottar.ness@ntnu.no; 3 Department of Public Health Science, Faculty of Landscape and Society, Norwegian University of Life Sciences (NMBU), 1430 Ås, Norway; linda.nesse@nmbu.no

**Keywords:** recovery, meta-synthesis, mental health and substance abuse, processual, ordinary life

## Abstract

Recovery, a prominent concern in mental health care worldwide, has been variously defined, requiring further clarification of the term as processual. Few studies have comprehensively addressed the nature of recovery processes. This study aims to explore the nature and characteristics of experiences of recovery as processual. The method used is a form of qualitative meta-synthesis that integrates the findings from 28 qualitative studies published during the past 15 years by one research group. Three meta-themes were developed: (a) recovery processes as step-wise, cyclical, and continuous, (b) recovery as everyday experiences, and (c) recovery as relational. These themes describe how recovery is intertwined with the way life in general unfolds in terms of human relationships, learning, coping, and ordinary everyday living. This meta-synthesis consolidates an understanding of recovery as fundamental processes of living in terms of being, doing, and accessing. These processes are contextualized in relation to mental health and/or substance abuse problems and highlight the need for support to facilitate the person’s access to necessary personal, social, and material resources to live an ordinary life in recovery.

## 1. Introduction

For about two decades, researchers at the Center for Mental Health and Substance Abuse (CMHSA) at the University of South-Eastern Norway have contributed to research in recovery and recovery-oriented practice. The many studies conducted over the years encompass a variety of descriptions, interpretations, and suggestions as to how “recovery” may be meaningfully understood and practiced. The rich empirical material accumulated in the studies of community mental health and substance abuse practices has been explored in a meta-synthesis addressing the research question: What are the characteristics of experiences and processes of recovery, and what are the experiences with recovery-oriented practice in mental health and substance abuse services? The findings from this meta-synthesis are presented in three parts: Part 1 addresses experiences of recovery, Part 2 focuses on how the processes of recovery unfold and materialize, and Part 3 concentrates on recovery-oriented practice. In this article (Part 2), the focus is on recovery understood as a process. This calls for a reminder of a discussion over several years on whether recovery should be understood as a process, as an outcome, or as a combination of both [1,2,3]

Davidson and colleagues argued that recovery as a process needs to be understood on its own terms and not necessarily linked to outcomes [2]. Rather, recovery as a process has to do with leading full lives in the face of mental illness and within traditional psychiatry, which is not ordinarily defined as an outcome. Broadly speaking, recovery as an outcome is derived from the perspective of clinical research and traditional psychiatry [4]. In this paradigm, recovery is typically referred to as “clinical recovery” and includes remission of symptoms and functional improvement, commonly through therapy and medication [5,6,7]. This is congruent with the biomedical model, which aims at treating the illness and not the person suffering from the illness, who is living a life in a social context [8]. Outcome-oriented recovery has been criticized for being solely individual-focused and paternalistic [9,10,11,12]. Critiques also address the disregard of people’s own efforts in their recovery [2,13], the everyday life context [14,15], and the possibilities and barriers to human rights and social participation [16,17,18]. Recovery viewed as a process is supported by reports from service users/survivors [19,20,21]. Recovery as processual involves both a personal and a social process and the relation between the two. It focuses on finding ways of living well either with or without symptoms or clinical problems [1,15,22]. Narratives of mental health recovery have increased the understanding of recovery as diverse, multidimensional, and non-linear, often considered in regards to social, political, and rights aspects [23]. Key social factors promoting recovery processes have been identified as empowerment and negotiating positive social identities, supportive personal relationships, and social inclusion [17,24]. Supportive relationships hold the potential to enable individuals to connect with the social world and to lead full and contributing lives as active citizens [25,26]. Price-Robertson and colleagues advocated relational recovery, which views recovery processes as inseparable from the social and cultural milieus from which they emerge [27]. Relational recovery addresses the need for recovery to grow beyond its roots in an individually-focused, non-contextual understanding in order to meet the complex realities, identities, and challenges faced by many people living with mental illness [27].

The person’s recovery process does not happen in a vacuum. Recovery as a process addresses the importance of expanding a focus solely on the individual to a focus on social and material conditions and on environments supportive of recovery [17,28,29]. In addition to direct work with clients, recovery-oriented practices should encompass collaboration with families, systems, and communities [30,31]. Tew highlighted the need to mobilize different forms of capital to promote recovery, i.e., economic, social, relational, identity, and personal capital [32]. In line with this, Rowe and Davidson emphasized the importance of contextual factors of recovery [33]. A recovery approach encompassing social and environmental factors may promote social capability in a sustainable way, supporting people to move forward from a current situation characterized by a sense of powerlessness and disconnection [17].

The literature supports the complex and various ways recovery is experienced, described, and understood [4,13,20,34,35]. Recovery as processual is also described with a multitude of meanings. Researchers have increasingly explored recovery processes from the perspective of persons with mental health and substance abuse problems. However, few studies have summarized the emerging evidence on recovery processes. With its overview of recovery research, this meta-synthesis aims to enhance understanding of the complexity of recovery processes. This aim is addressed by conducting a meta-synthesis of papers on the topic of recovery by a research group dedicated to the topic of recovery rather than carrying out a general meta-synthesis of papers published at large. This approach was selected to focus on a meta-synthesis of papers that have a specific qualitative orientation in interpretive phenomenology. Clinical practice and future research may benefit from an up-to-date systematic review of how recovery as processual may be described and understood.

## 2. Method

### 2.1. The Research Context

Recovery has been a key area of research at the CMHSA since the early 2000s. The Center has a specific focus on collaborative research methodologies with people with lived experience, family members, and practitioners. The CMHSA engages people with a variety of experiences and a wide range of knowledge as key partners in research. Our recovery research has from the outset focused on subjective experiences, relational aspects, everyday life experiences, and the impact of material and social conditions as well as recovery-oriented services, community-based support systems, and peer support work. Furthermore, the Center conducts research in dialogical and collaborative practices and child and adolescent issues. The researchers have varied professional backgrounds in the health and social care sector and a wide range of clinical practice experiences in addition to lived experience. The Center has expertise in qualitative, quantitative, and triangulation/mixed methodologies.

### 2.2. Qualitative Meta-Syntheses

The method applied in this paper is a form of qualitative meta-synthesis. The term qualitative meta-synthesis has various meanings, refers to a variety of approaches, and is often used in systematic review studies. The qualitative meta-synthesis in this paper is in line with the first kind of synthesis identified by Sandelowski and colleagues, which referred to integrating the findings from multiple qualitative studies within a program of research by the same investigators [36]. The purpose of this approach in the present paper is to explore how recovery is described in empirical research at the CMHSA, addressing the research question of “How is recovery described in empirical research at the CMHSA in the period 2005–2020?” The objective is to arrive at a theoretically meaningful synthesis about recovery as experiences, processes, and service orientations through the integration and comparison of the qualitative empirical material accumulated by CMHSA researchers in their studies of community mental health and substance abuse practices. The procedural steps adopted reflect the seven steps identified by Noblit and Hare [37] for meta-ethnography, which consist of (1) getting started, (2) deciding what is relevant to the initial interest, (3) reading the studies, (4) determining how the studies are related, (5) translating the studies into one another, (6) synthesizing translations, and (7) expressing the synthesis.

The publications included in this meta-synthesis were written by CMHSA researchers, whose research orientation as a group is recovery and recovery-oriented practice. The focus of our synthesis was recovery experiences, processes of recovery, and recovery-oriented mental health and substance abuse practices. The first four steps of Noblit and Hare’s method have been well established within the group. This qualitative meta-synthesis thus encompasses the last three steps, namely translating the studies into one another, synthesizing those translations, and expressing the synthesis. Meta-ethnography and meta-syntheses in general are oriented to “synthesizing” researchers’ interpretations of qualitative data in original studies, which are social constructions “built into accounts of methods, in the theories used, in the researchers’ worldviews” ([38], p. 3). However, this meta-synthesis did not have to deal with the issue of consolidating different perspectives or worldviews. It began with the prior knowledge of our perspectives, methods, and worldviews, which align with the epistemological stance of a phenomenological-interpretive and critical perspective. For the fifth step of translating the studies into one another, the themes and concepts from each study with their descriptors were identified, compared, and contrasted, which also involved reflections on relevant literature. Based on the results from the fifth step, the sixth step involved meta-synthesizing the themes and concepts regarding recovery experiences, processes, and practice orientations. Thus, this step involved using the researchers’ judgment and creativity, which is critical in qualitative synthesis [39]. The synthesis of themes and concepts found in these publications involved consolidating similar themes and specifying them into meta-themes by comparing the themes and their meanings. Some themes extracted from individual publications were also specified as meta-themes when considered critical in providing the meanings of recovery experiences, processes, or practice orientations. The seventh step of the meta-synthesis, “expressing the synthesis”, involved systematizing the results of the meta-synthesis.

**Figure 1:**
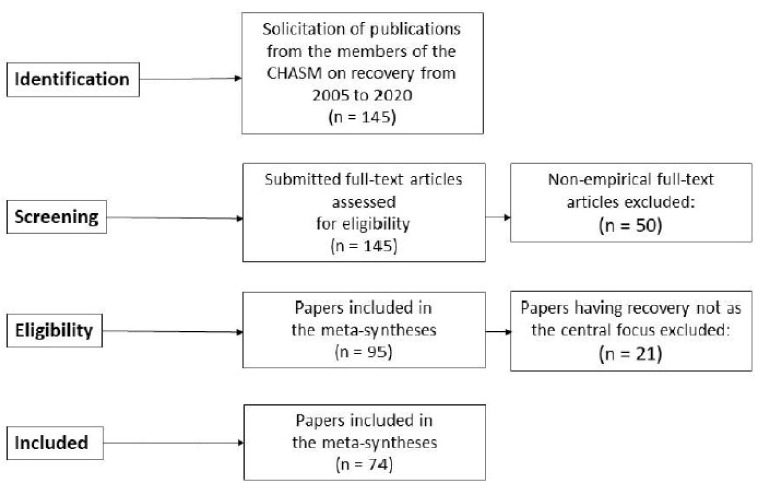
Flow chart of the process of gathering the publications for the meta-syntheses.

Figure 1 shows the steps taken by the research team for the meta-syntheses for Parts 1, 2, and 3, using a PRISMA flow diagram. The details of the steps followed in assembling the database for this study are somewhat simplified because the publications included in these meta-syntheses were those of the members of the CMHSA research team.

The steps of collecting, reviewing, and analyzing the papers were as follows. A core research group of five CMHSA researchers was established to be responsible for the meta-syntheses and writing the results for publication. All 20 researchers in CMHSA were then invited to contribute to the study and requested to submit their publications to the core group. Sixteen researchers accepted the invitation. The inclusion criteria for the papers were: (a) the paper’s central focus should be recovery for people with MHSA problems, (b) the paper should address recovery as lived experiences, and (c) the paper should have applied a qualitative method, (d) the paper should have been published from 2005 to 2020. We also invited the researchers to include other papers that might be relevant to the topic. Based on these inclusion criteria, we first excluded papers that have applied a quantitative method or are without empirical contents including those that were theoretical presentations. We then excluded those papers that dealt mainly with mental health problems as clinically oriented phenomena rather than focusing on recovery. The languages included were English and Scandinavian languages (Norwegian, Danish, and Swedish). A total of 145 papers were submitted.

These papers were reviewed by the core research group in relation to the research question, resulting in the final selection of 95 empirically oriented papers. Each of these papers was systematized by using a data extraction form inspired by Critical Appraisal Skills Program (CASP) for quality appraisal in qualitative evidence synthesis [38]. We did not use CASP to evaluate the papers, but in order to assess the key characteristics of the papers included in this study. The studies employed qualitative methods, mostly focus group and in-depth individual interviews with research participants who where service users, family members or significant others of service users, and professionals. The analytical methods used in these studies were descriptive and/or interpretive. An examination of this set of publications by the core group resulted in a division of the material into three broad topic areas: (a) recovery as personal and/or contextual experiences, (b) recovery as processual, and (c) recovery-oriented services and practice. Therefore, three meta-syntheses were performed using these data. There were 28 papers in the topic areas of recovery as personal and/or contextual experiences and as processual, and 46 papers in the topic area of recovery-oriented services and practices. We planned to write three papers, each focusing on a meta-synthesis of one of the three topic areas. All 28 papers were the basis for the two meta-synthesis processes applied to address recovery as experiences and recovery as processual presented as Part 1 and Part 2.

The 28 papers mentioned above formed the basis of two meta-syntheses presented in the papers as Part 1 and Part 2. This paper deals with the meta-synthesis of recovery as processual.

## 3. Results

This meta-synthesis presents the results regarding processes of recovery elicited from the papers listed in Table 1.

The included papers are listed chronologically in Table 1. The table includes brief descriptions of methods used, research participants, and important themes describing and exploring dimensions of recovery.

The studies were conducted in the context of community mental health and substance abuse practice. They included participants with experience of diverse mental health and substance abuse difficulties, both acute and long-term, who had received a variety of mental health and substance abuse services. Six of the studies were based on a multi-national collaboration between Italy, the USA, Sweden, and Norway. The other twenty-two studies were based solely in a Norwegian context.

The meta-themes in this presentation are based on a consolidation of similar themes in the included papers, followed by a synthesis of the themes and their meanings into overarching meta-themes across the set of included studies. The process of synthesizing the themes from these papers began with the reading of the included papers by the core research team individually and then discussing similarities and varieties among them. This was followed by identifying those themes which share common meanings. The next step involved consolidating those thematic ideas sharing common meanings into major themes in the meta-synthesis. Because there was only one paper each with data from healthcare providers and users’ relatives, the themes presented in these two papers were considered in relation to the ways they supported the themes emerging from the data obtained with service users. The synthesis aims to capture all-encompassing patterns and themes, but also variety and diversity as important factors in how recovery as person-context experiences and dynamics is described.

The meta-synthesis yielded two overarching patterns describing recovery in empirical research: (a) recovery as person-context experiences and dynamics and (b) recovery as processual experiences. This paper will explore only the second pattern. The first pattern is described in the paper referred to as Part 1.

Recovery as processual emerged from this analysis as an overarching pattern reflecting recovery as a process continuously making, remaking, and unmaking itself, in a constant interplay between individuals and the contexts of their lives. Rather than emphasizing outcomes, recovery as processual embraces the journey with its unpredictable detours in multifaceted landscapes. Recovery as processual is fluid and intertwined with the complexity of everyday life. It is not within the sphere of the controllable and measurable aspects of life.

Recovery as processual can be understood as having three meanings: (a) recovery processes as step-wise, cyclical, and continuous, (b) recovery as everyday experiences, and (c) recovery as relational.

### 3.1. Recovery Processes as Step-Wise, Cyclical, and Continuous

Recovery is not an orderly and linear process following predictable forward-moving steps to expected outcomes. Life is unpredictable for most people, not just for those in recovery. Recovery occurs in steps and in cyclical and continuous processes. It involves movement back and forth, and vacillation between the known and the unknown in ever-changing landscapes. In short, recovery takes place within life’s complexity, variability, and unpredictability. Recovery processes as step-wise, cyclical, and continuous can be understood as: (a) a process involving steps forward and steps backward and (b) a process involving all aspects of one’s life.

#### 3.1.1. A Process Involving Steps Forward and Steps Backward

The experience of moving forward at one moment and backward at the next can be understood as moving between past, present, and future and as interruptions in linear forward progress. Recovery is not a stable and coherent concept, but has been described as a unique process that differs among individuals [40]. The recovery process has been depicted as relating to one’s past as well as one’s present and future, often simultaneously, in order to give meaning to the lived experiences of the particular situation [53,64]. Veseth and colleagues [53] found that a state of not knowing what was happening as mental health problems intruded into everyday life led to the frightening experience of not recognizing oneself. Uncertainty and confusion put the normal movement of stepping forward in life on hold. Steps towards understanding one’s difficulties as a battle with mental health problems, towards acceptance about how this was affecting one’s life, and finding out how to deal with these challenges was an active process. Veseth and colleagues [53] also described how giving meanings to the lived experiences of the particular situation and to the specific symptoms was critical for the recovery process. The need to give meanings was a drive and also a challenge in the process of recovery. Being attentive to signs of ups and downs also operates in a dynamic relationship with holding on to steadiness [53]. When the world is spinning round, finding an anchor of stability is important in taking care of oneself and in promoting recovery [51,53,58].

Communicating one’s experiences with professionals, family members, and others, and trying out different approaches to managing symptoms and clinical issues due to mental health problems were part of the individual’s active process that initiated the step forward. Semb and colleagues [64] found that young adults with co-occurring mental health and substance abuse issues (MHSA) underlined the need to accept a person’s life story of problems in the past as well as current problems such as capacity for work or study and diminished dreams for the future. Young adults with MHSA were, more or less, left on their own in this struggle with past and present problems. Providing safety for individuals when they step back and hope for possibilities when they step forward requires a great deal of social and professional support [53,64].

#### 3.1.2. A Process Involving All Aspects of One’s Life

Recovery as a process involving all aspects of life requires an integrated understanding of recovery, which includes considerably more than symptom management. The recovery process involving all aspects of one’s life is concerned with becoming an ordinary member of society, thus fulfilling all the expected obligations and having a good quality of life. The key aspect of this process is the alignment of the one who enters with those already there, both symbolically and concretely. For the person in recovery, an integrated understanding of recovery also includes a focus on developing a new identity, having a social life, belonging to a local community, and having hopes and dreams for the present and the future [47,57]. Semb and colleagues found that young adults’ experiences of belonging and inclusion in local communities were challenged by their personal experiences of belonging to an “outsider life” [64]. Their previous experiences, such as being out of school and work, struggling with symptoms and other experiences specific to mental health problems and substance abuse, were viewed as irrelevant or invalid in mainstream society. Furthermore, being stigmatized and marginalized by others in the mainstream world limited the pursuit of a “normal” social life. On the other hand, adapting to the accepted rules in mainstream society made belonging easier. Semb and colleagues stated that in addition to various social and mental health initiatives, expanding the framework of what is viewed as a valid or legitimate life could support individuals in their recovery process, maneuvering them towards new identities and belonging in local communities in mainstream society [64].

### 3.2. Recovery as Everyday Experiences

Recovery as everyday experiences refers to dynamic processes taking place in the context of everyday life, including the ways people integrate personal resources as well as material resources and social issues. It points to recovery processes as interwoven with the person’s everyday life, not taking place outside of life in general. This meta-theme encompasses two sub-themes: (a) struggling to achieve or remain in a normal, ordinary life, and (b) accessing resources, possibilities, and enjoyment.

#### 3.2.1. Struggling to Achieve or Remain in a Normal, Ordinary Life

The basis for this process as evidenced in our papers is the concept of “normal” determined by the participants of the studies themselves rather than the standardized notion of the concept. Therefore, all references to “normal” are based on assessments by clients themselves, which usually vary in terms of emphasis, depending on their experiences of mental health and/or substance abuse problems. Struggling to achieve or remain in a normal, ordinary life is critical to recovery processes and reflects the heart of recovery; it means living a life in society like everyone else, with its ups and downs, pleasures and sorrows. It refers to having responsibilities, contributing, mattering to others, being appreciated, growing and thriving, and having a meaningful life. Borg and Davidson found that normality has very specific meanings, such as spending time in ordinary environments with ordinary people and accomplishing the taken-for-granted activities of daily life which can be difficult due to mental illness [14]. Having a normal, ordinary life included being situated in ordinary social settings and fulfilling ordinary roles in family and social life such as being a family member and having a job [14,40,48,52]. Managing the lingering effects of mental illness was also an important aspect of regaining normalcy as was developing friendships and romantic relationships [40]. Having a safe and comfortable home and adequate financial resources for participation in ordinary activities was also critical to achieving and maintaining an ordinary life [14,40,42,45]. Furthermore, developing and maintaining meaningful routines, such as having a job or participating in other activities on a regular basis, physical activity, adequate sleep, and stable meals supported normality and reduced stress and loneliness [41,67]. Davidson and colleagues found that a less acknowledged part of the recovery process is the person’s acceptance that even a “normal” life has its problems, its ups and downs, joys and disappointments, regardless of mental health problems [40]. However, dealing with this for persons in recovery requires conscious efforts because either they have been unable to maintain a normal, ordinary life due to the difficulties inherent in MHSA or there are social and contextual forces that tend to pull the persons toward the outer bounds of a normal life.

#### 3.2.2. Accessing Resources, Possibilities, and Enjoyment

Opportunities and access to social and material resources, possibilities, and enjoyment are part of everyday life and intertwined with personal and social recovery processes. Resources, which include personal, material, contextual, and environmental factors, provide opportunities for recovery [59]. It is crucial that mental health professionals give priority to making sure that state benefits for individual citizens are partly used to support recovery processes [14]. Professionals and the community need to see the people beyond their diagnoses, accepting them as fellow citizens [56,57]. Focusing on enhancing and strengthening access to resources, as a counterbalance to focusing on problems and limitations, enabled participation and engagement in social and cultural activities, and the realization of hopes and dreams [40,41,43,56]. Opportunities to participate in meaningful activities offered enjoyment and pleasure and boosted self-esteem [40]. Having fun and enjoyment were valuable experiences in everyday struggling and coping, supporting the recovery process [14].

### 3.3. Recovery as Relational

Recovery processes unfold within a social and interpersonal context. Social relationships play a central role in recovery processes and include relationships with both professionals and “ordinary” people. In our analysis, recovery as relational was synthesized into three themes: (a) developing and maintaining supportive relationships, (b) accessing supportive environments, and (c) engaging in relational hope.

#### 3.3.1. Developing and Maintaining Supportive Relationships

The key process in the perspective of recovery as relational is developing and maintaining supportive relationships in recovery. Recovery takes place in relation to other people in one’s environment. A variety of relationships beneficial to the recovery process are identified as “supportive relationships”. Topor and colleagues stated that relationships contributing to a person’s recovery were found among friends, family members, and professional helpers [49]. Further, relationships with peers allowed for mutual understanding and recognition of the person’s recovery efforts [60,63]. The categories of “friendship” and “professional helpers” were not always clearly distinctive. Professionals could be “friends”, where this description indicated qualities in the relationship that are recognized in friendship [44]. Crucial relational qualities beneficial for recovery were the experience of being viewed as equal, being understood and accepted, being cared for, and receiving kindness [40,43,65,66]. Being there for the person and standing by the person in good times and bad, offering practical support, and advocating for the person’s needs and rights contributed to recovery [40,44]. Supportive relationships were not dependent on the helper’s formal education or training. Instead, they depended on the ability to relate to the individual in recovery as a person, not as an object [44]. Supportive relationships enhancing recovery were recognized as collaborative and dependent on a mutual experience of seeing each other as a person [14,64]. Collaborative relationships facilitated experiences of belonging and empowerment because they allowed individuals to make decisions in their own best interests [40,66].

Significant and supportive others were interwoven within the context of the individuals’ lives [43]. The variety of social relationships providing firm support indicates the importance of including people outside professional services in recovery processes. Ogundipe and colleagues pointed out the need for more communal and contextually oriented approaches in mental health services [66].

#### 3.3.2. Accessing Supportive Environments

One’s environment is the bedrock of resources that are critical, meaningful, and helpful for one’s living. Recovery as the process of living thus has to draw on the necessary and supportive resources from one’s environment. Accessing supportive environments in recovery is therefore dependent on the person’s relationship with these environments. To understand supportive environments in the context of recovery processes, it is necessary to view mental health problems and social challenges as intertwined and as mutually affecting each other. Davidson and colleagues [40] found that support in managing symptoms of mental illness was provided in formal mental health services and in participation in peer support groups or by searching for information in self-help material or on the Internet. People were actively searching for and trying out strategies that could reduce and help them to manage their symptoms [40,51]. These strategies included family support, support from professionals, medication, and knowledge of one’s illness and recovery process [40,49]. Another element of supportive environments was reengagement in ordinary activities, such as work or school, and participating in naturally occurring social and recreational activities [40,43,67]. Understanding recovery processes requires a broad perspective on the meaning of supportive environments. A supportive environment is contextual and incorporates a variety of life experiences including recognition of struggles in recovery processes and strategies for overcoming them. Furthermore, both problems and successes should be recognized as multifaceted, which requires an understanding of a supportive environment that embraces deep insight into the contextual dimensions of recovery.

#### 3.3.3. Engaging in Relational Hope

In our analysis, hope appeared to be a significant and integral aspect of recovery processes. Hope can be understood as a relational phenomenon that can be both strengthened and threatened in relationships. Hope showed itself to be strong and fragile and solid and shifting [61]. Nourishment of hope necessitated a reliable person with faith in the potential of the person in recovery [54]. Hope was inherent in the process of accepting one’s life situation and finding realistic goals for the present and the future [53,61]. The goals could be small and concrete, related to everyday coping, while future goals could be more extensive. Support from others, both from inside and outside mental health services, facilitates hope for a good and meaningful life despite mental health problems [55]. Being seen and acknowledged in social relationships created hope and helped people to verbalize options and solutions in difficult life situations [46]. Hope offered the possibility for the persons to make changes in their lives [54]. Capacity for change was established when hope was supported [61]. Hope was valued as a process and a way of living, not as a goal to be achieved. Clinicians need to be aware that sometimes maintaining hope as a way of living may be more important than seeing a particular hope fulfilled [50].

## 4. Discussion

Based on this meta-analysis, recovery is understood as a process that is indivisible, concrete, and temporal, revealing the central position of the person in his/her specific social environment. Recovery processes can be viewed as dynamic, encompassing “something going on”, growing and developing, and for a purpose (https://www.merriam-webster.com/dictionary/process (accessed on 20 March 2021). Recovery processes are characterized as non-linear, stepwise, and cyclical, and reflect how life in general unfolds in terms of human relationships, learning, coping, and ordinary everyday living. In this section, three meta-themes that resulted from the meta-synthesis are reframed as more generic processes of being to encompass the non-linear, stepwise, and cyclical nature of life itself, doing as the way of carrying on with everyday life, and accessing as representing people’s dependence on others and their environment for support, co-existence, and life satisfaction.

### 4.1. Recovery as Being

Recovery takes place within life’s complexity and unpredictability. Based on the findings, we understand *recovery as being* as related to the temporal unfolding of meaning, being well in everyday life, belonging, and accepting. These findings expand on previous attempts to conceptualize the existential aspects of the process of personal recovery in serious illness.

In their discussion of the roots and developments of personal recovery, Hummelvoll and colleagues argued that the process of personal recovery in mental illness is characterized by existential or spiritual concerns, evolving around the idea of being in recovery [68]. This existential element suggests that being has to do with authenticity, understood as an essential part of both being human and becoming a human in a unique sense. This existential and yet processual feature of recovery is constituted not only by hope, but also by interaction with the self, others, and the social world [62,68]. This somewhat synthesized description of the process of personal recovery and its existential features echoes previous research conducted by Davidson and colleagues [69]. In their outline of the distinctive features of personal recovery, they argued that “being” characterizes personal recovery processes in at least four partially overlapping ways: (a) redefining one’s sense of self and assuming control of one’s life in general, which includes the ability to manage one’s symptoms by using treatment options of one’s choice to bring the symptoms under control, (b) being involved in meaningful activities, which includes occupying social roles such as spouse, worker, student, taxpayer, friend, and someone actively and responsibly contributing to a community, (c) being supported by others, which involves returning to work and/or mending broken relationships and thus becoming interdependent with others in the community and having supportive others around one, and (d) becoming an empowered citizen by regaining control over one’s life and emphasizing one’s right to live, love, participate, and take on responsibilities [69].

Recovery as “being” in this study emerges in the stepwise and cyclical nature of personal recovery processes. One of the key existential features of people’s being in ordinary life situations is unpredictability, but people in general are able to cope with this and move forward without doubling back to earlier states. However, a person with MHSA in recovery is more likely to face complex unpredictability that is configured with the vicissitudes and variability associated with such problems even when in treatment. This means that the process of “being” in recovery for MHSA clients does not move forward linearly, but often has to double back in a cyclical and stepwise manner to deal with vacillations and complexities. This involves learning how to integrate meanings, successes, and failures from the past with those encountered in the present and projected for the future. It mirrors how people living with mental illness previously have been known to redefine their sense of self, re-assume control in life, and manage symptoms through better or harder times. The existential dimension of recovery also emerges through the description of recovery as a process involving all aspects of one’s life. This feature aligns with the characterization of recovery as a process in which the person experiencing severe mental distress carries out or participates in meaningful activities that emphasize the right to live and love, and have the potential for the person to contribute in the community alongside others. Equally, the descriptions of how the process of recovery often involves a struggle to achieve or remain in a normal, ordinary life are similar to the importance of occupying normal, social roles in family life, friendships, at work or as a citizen. Supportive relationships and mended relationships established in recovery convey hope and enable the person to live a meaningful and independent life in the community and have access to support from others.

Human existence has been the subject of philosophical discussion by several scholars such as Kierkegaard, Sartre, Taylor, and Heidegger. For Heidegger [70], human existence is the temporal unfolding and creating of a life course and is about ordinary everydayness. Everydayness means being caught up in the processes of practical affairs of life and acting in an ordinary pre-reflective way. Drawing on Heidegger’s perspective on being, recovery-as-being involves the existence of nature and other human beings. At the same time, everydayness means being oriented towards the future and realizing one’s potentials until death completes the self and the life course. Recovery as being is not a normative recommendation about how people should live.

### 4.2. Recovery as Doing

The results of this meta-synthesis show that doing recovery is about participating and contributing through ordinary everyday activities within a social context in which a person feels valued. Participating and contributing are made possible by a journey of personal transformations to new identities in which a person develops the capacity and is provided with opportunities to participate in meaningful routines and activities while also coping with the lingering effects of mental illness and encountering the normal ups and downs of life. This personal journey takes place within a social context [71]. This implies that social relationships and networks constitute the medium through which personal transformation becomes possible. Opportunities to care for others and for community participation may be particularly important for recovery [72].

A journey of personal transformation may involve (re)discovering a degree of self-efficacy, i.e., a combination of beliefs and abilities that underscore one’s confidence in taking the initiative and starting to influence one’s own situation [73], such as when recovery processes involve disconnecting from certain relationships and setting boundaries. Additionally, the development of self-efficacy may assist in overcoming experiences of profound personal and social dislocation, oppression, or social defeat and lay the foundations for a rewarding and meaningful life, even when having to manage certain ongoing distress “symptoms”, although these may often recede if people’s circumstances become more conducive to well-being and processes of recovery [13]. All the experiences which are part of mental distress and suffering are part of the experiential dimensions of ordinary life. Recognizing this dimension of normality is beneficial to self-efficacy and assists in developing a new identity in recovery processes.

Relationships are vital building blocks in recovery processes as they shape identity and promote or hinder well-being. Understanding self-efficacy more broadly as the ability to develop “power together” with others may create social opportunities or provide mutual support, which is important in the recovery process [32]. As highlighted in several of the included studies, recognition by and connectedness with other community members, such as friends, family, professional helpers, and peers, promotes recovery processes [14,22,46,49,60,63,64,66]. Through connectedness, a sense of mattering to others is developed [74]. Narusson and Wilken argue that recovery processes should offer “hope and empowerment emerging from the contact between individuals and the social and cultural milieus in which they are embedded” [75]. This meta-analysis supports the notion that *doing* recovery is both a personal and a social process and the dynamics between the two. It affirms that recovery processes involve having a normal, ordinary life situated in common interpersonal and wider social settings and engaging in a range of social domains such as family life, and in arenas of productive activity and recreation, and having the feeling that one belongs and has a place within one’s social situations and community. It may also involve challenging a range of exclusionary barriers to such engagement as well as co-creating new opportunities where existing opportunities may be limiting.

### 4.3. Recovery as Accessing

This meta-analysis shows that people in recovery desire to participate in ordinary life. Participating in ordinary life requires not only the individual work of recreating a new identity but accessing and being able to participate in a community. People in recovery seek to find ways to experience belonging in their neighborhood and larger society, trying out new roles and working to achieve what they consider a normal, ordinary life. Accessing nurturing environments such as friendly and welcoming places, supportive locations, and safe communities is essential in the efforts to belong, create a new identity, try out new roles, and lead a normal ordinary life [21,76]. Accessing aims for knowledge and understanding as well as practical help and supportive relationships. Gaining local knowledge and insights into what the community has to offer is essential, such as how to access safe housing, how to access opportunities for work, and how to participate as a member of the community. These are important skills that can enrich the recovery process. The availability of material, social, and financial resources is a precondition for the possibility to be in an ordinary environment with ordinary people.

Furthermore, this meta-analysis shows the importance and centrality of relationships and belonging in recovery. People in recovery processes need to engage in social relationships and experience belonging in social groups and communities. The people in this study felt that their past identities made social inclusion difficult. Studies have described stigma and discrimination by others in mainstream society as barriers to social inclusion [18]. Such social exclusion is experienced as hindering social belonging, with issues such as marginalization contributing to the feeling of being an outsider and different from others [64]. Furthermore, lacking access to necessary material resources in life may also contribute to social exclusion. Part of being ordinary is to understand that people in general have the experiences and challenges that constitute what are viewed as mental health problems and substance abuse issues. All the experiences involved in mental suffering are part of the experiential dimensions of ordinary life. However, the marginalization, stigmatization, and labeling that may happen to persons with MHSA problems often lead to the denial of access to meaningful and necessary resources in society. Thus, persons in recovery have to make an extraordinary effort to gain or regain such resources.

It is hard to “recover” when faced with unemployment, poor finances, inadequate housing, and exclusion from ordinary social life. The availability of material resources is a prerequisite for being in an ordinary environment with ordinary people. Thus, there is a circular dynamic in which belonging and having social resources in an inclusive community must coexist with having and being able to access adequate material resources. Participating in a social network and an inclusive larger community with a new identity and adequate material resources is what ordinary life is.

### 4.4. Three Fundamental Processes in Recovery

The generic processes of being, doing, and accessing, representing the themes identified in this meta-synthesis, can be considered the fundamental processes in recovery. These processes, therefore, involve three intertwined areas: being as the ways the person in recovery establishes/re-establishes identity in the ever-changing and fluctuating course of life, doing as how the person participates in and maneuvers everyday life and tries to find meaning and fulfilment, and accessing as how the person seeks out resources and relationships in order to find pleasure and deal with the demands of life. These three areas support the arguments for recovering citizenship of Rowe and Davidson [71]. Recovering citizenship is based on the logic that access to fundamental resources is a necessity for people’s recovery processes. Furthermore, recent literature on recovery capital argues for a new paradigm in mental health that is oriented towards enabling the development of personal efficacy and social capability [77,78]. Recovery capital includes aspects such as financial capital, social capital, personal capital, relationship capital, and identity capital, all of which may be important in assessing a person’s potential. The three areas of recovery processes (being, doing, and accessing) identified in our meta-synthesis enhance our understanding of the interrelationship of personal efficacy and social capability. Our findings also provide a way to advance the five themes identified by Dell and colleagues in their meta-synthesis of 25 systematic reviews on recovery by specifying the ecologically oriented features of recovery [79]. The themes generated in that study were (a) recovery as a process of overcoming despair to realize a positive sense of self and well-being, (b) environmental requirements necessary for recovery, (c) the role of autonomy, control, and personal responsibility, (d) the importance of social support and meaningful activities to the development of a sense of belonging and purpose, and (e) developing acceptance of one’s illness and insight into how to establish and maintain wellness ([22], p. 7–8). The general tenet of these themes emphasizes the interplay between the self and the environment during recovery, which supports the processes of recovery. The three fundamental processes in recovery identified in our study thus specify how a person can attain “a positive sense of self and well-being” which Dell and colleagues identified as the key feature of the recovery process [79].

### 4.5. Limitations

Although this meta-synthesis of the studies by one research group (the CMHSA) has strengths in terms of its findings being coherent and integral, framed by the perspective of the research team, it also presents limitations because of the study’s orientation to a specific perspective. The authors (i.e., the core researchers for this meta-synthesis) had intimate knowledge of the intentions, contexts, and orientations of the authors of the papers in this meta-synthesis. This is a strength because of our in-depth understanding regarding the papers and also could be a weakness due to alliances and bias. The findings are limited by the ways the data were analyzed in the original studies and the interpretive perspective adopted in the analysis. It is possible that a more comprehensive, diversified understanding could have been gained by a meta-synthesis of studies with a greater variety of perspectives and analytic methods. However, the richness of the findings in the study adds to our knowledge of the processes of recovery, providing in-depth understandings gained from an analysis that took into account the perspectives of research participants who were mostly users and their families. A related limitation concerns the generalizability of the findings in characterizing the processes of recovery, since the studies were mostly carried out in Scandinavian countries. However, if we generally accept the idea that processes of recovery are inherently embedded in universal human processes, albeit in the context of mental health and/or substance abuse, the findings enrich our understanding of the processes of recovery.

## 5. Conclusions

This meta-synthesis about processes of recovery has identified three key processes: being, doing, and accessing. These processes can be viewed as fundamental processes of living. However, these processes of living are contextualized in recovery in relation to mental health and/or substance abuse problems. Therefore, the processes of being, doing, and accessing take on special characteristics determined by the constraints, experiences, and demands associated with mental health and/or substance abuse problems. This means that the processes of being, doing, and accessing in recovery take place in the context of difficulties such as accentuated uncertainty, unpredictability, increased vulnerability, and loss of ordinary resources and social networks. These problems are inherent in mental health and/or substance abuse as well as in the context of non-ordinary or extra-ordinary demands such as clinical symptom management, management of chronicity, and recidivism faced by the clients involved. Such special characteristics of these processes require people in recovery to develop new or revised ways of engaging themselves in living and to have specific kinds of support that can enhance their engagement in the processes. One important issue is to develop and refine specific types of general and professional support that can enhance positive experiences in recovery processes. These themes emerging from our study suggest the extent to which recovery processes are integral to the processes of everyday living. This suggests that the recovery practice has to be framed and designed in the context of everyday living. Since the processes of recovery identified in this study are inherently embedded in processes of living, further research should address the relationship between living and recovery from a process perspective in order to understand the issues of continuity, learning, and habits.

## Figures and Tables

**Table 1:** Included papers in the meta-syntheses.

Publications	Research Question(s)	Methods	Research Participants	Themes and Meanings
[40]	Explore processes of recovery in psychosis	Narrative and phenomenological approach with individual interviews	Twelve adults with experiences of recovery in psychosis	The person’s determination to get better, establishing a degree of self-control, and struggling to achieve a normal life in dealing with problems The need for material resources and a sense of home, and the importance of going out and engaging in normal activities Roles of formal/informal health systems in terms of the benefits of medication, involvement in mutual support/user groups, and participation in various psychosocial interventions The need to be accepted and to accept oneself as a normal person who exists beyond the psychosis; the impact of stigma and discrimination, and the importance of having one’s rights respected and returning to a meaningful social role through work and/or positive relationships outside of the formal mental health system The roles of social and cultural factors for the persons in terms of opportunities and support offered
[41]	To describe service system contexts in which the informants lived and received services and support	Phenomenological narrative interviews	Twelve persons in recovery	Roles of home, significant others, and coping strategies being interwoven in the context of individuals’ lives and personal recovery journeys.
[42]	How do people in recovery from psychosis develop and accept their role in society and where does that take place?	Qualitative interviews	Twelve adult service users in recovery from psychosis	Material resources in terms of their practical importance in daily life and their immaterial meanings such as emotional comfort Having a home meaning having a place for growth and development, a place of control, an opportunity to balance privacy and social life, and a place to long for and dream about.
[43]	To identify community settings that appear to foster recovery, as well as the mechanisms through which this takes place.	Qualitative individual interviews	Persons in recovery from psychosis	Involvement across various community settings can establish more beneficial and lasting understandings of the self. Being understood and accepted Fun and enjoyment Role shifting Meaningful routines Employment Spirituality Esteem Anger as a mechanism of empowerment and change Integrative aspects
[44]	(A) Can other people contribute to the recovery process? (B) If so, which people? (C) ©According to the informants, what do these people do that contributes to the recovery process?	Qualitative interviews	Twelve persons in recovery	Social relationships play key positive and negative roles in recovery processes A beneficial relationship is not dependent on the helper’s formal education or training Beneficial relationships are characterized by: (a) standing by the person with continuity, (b) being bearers of hope, (c) demonstrating through being there that the person is more than his/her illness, and (d) being there for the person in recovery, including providing practical support, intervening as advocates and lobbyists. Key characteristics of helping in recovery processes: (a) Being there for the person in recovery, (b) helping by doing more than expected, and (c) helping by doing something different than what was expected.
[45]	How do people in recovery from psychosis develop and accept their roles in society and where does that take place?	Qualitative interviews	Persons in recovery from psychosis	Social barriers to recovery: Stigma (and self-stigma) Being different (labeled) Exclusion and stigma (from normal social life, locked into role of mental patient) Social pathways to recovery: Self-advocacy Being in supportive social environment Finding new bonds and new roles Working and studying, thus enabling new roles and statuses Participation and citizenship with a sense of belonging
[46]	How is meaning constructed in narratives of suicidal behavior?	Phenomenological hermeneutic approach with narrative interviews	Four adult males receiving substance abuse services	The meaning of living with suicidal behavior as a movement between different positions of wanting death as an escape from pain and hope for a better life: the meaning of relating the meaning of reflecting the meaning of acting
[47]	To explore recovery within the context of the person’s everyday life	In-depth individual interviews	Thirteen adults in recovery	Being normal Just doing it Making life easier Being good to yourself
[48]	To identify and discuss the role that work plays on the road to recovery for people with severe mental illness, particularly those diagnosed with psychosis.	Phenomenological approach with in-depth individual interviews	Thirteen adult users with mental health problems	Being and becoming: an active worker not a passive patient Belonging in an ordinary working life Balancing—not too much, not too little Believing in oneself—the importance of supportive and flexible environments
[49]	To broaden the individual perspective on recovery by describing additional aspects of the journey that involve the contribution of others and various social factors and elements that can facilitate or impede inclusion in community life.	Qualitative individual interviews		The contribution of others, including friendship, families, and professionals Social factors including home, money and employment Structural recovery, including the need for recovery knowledge, including recovery of others and recovery of the services
[50]	How meaning is constructed in narratives of hope by persons that have recently engaged in suicidal behavior.	Hermeneutic-phenomenological approach using semi-structured in-depth interviews	Twelve adult patients admitted for overdose of medication	Relational hopes for life and death The meaning of hopes for life—hope in the context of relationships The meaning of the act of hoping projected as definite or indefinite hopes in terms of “stop or not,” “a limit or not,” and “a specific agency or not”
[51]	What do individuals with bipolar disorder do to promote their own recovery and what challenges do they meet?	Hermeneutic-phenomenological approach with individual in-depth interviews	Thirteen persons with bipolar disorder	Handling ambivalence about letting-go (i.e., accepting) of manic states Finding something to hang on to when the world is spinning around Becoming aware of signals from self and others Finding ways of caring for oneself
[52]	To understand the role of work in recovery from bipolar disorder, and to understand how people with such disorders deal with work-related challenges	Hermeneutic phenomenology and reflexive methodology	Thirteen adults with experience of bipolar disorder who are receiving or have received treatment	Meaning and structure provided by work involving a variety of activities including the job Helpful roles and contexts outside illness provided by work—roles and contexts in which clients can use their skills, feel needed and contribute Making work possible with support and help from others in one’s network Cost of working too much suggests work-rest balance; working too hard associated with clients’ initial episodes of mental health problems
[53]	Explore first person perspectives on identifying a bipolar disorder: how do individuals experience the process of discovering that they have a bipolar disorder? What does it mean for the person to find out that their symptoms and distress are in line with descriptions commonly seen as a severe mental illness?	Hermeneutic-phenomenological approach with individual in-depth interviews	Thirteen individuals with recovery experiences	Three phases of recovery: (a) “uncertainty and confusion” through (b) “grasping the novel and unusual experiential states” to (c) “giving meaning to the lived experiences of intense ups and downs”.
[54]	How do persons with co-occurring mental health and substance use problems (MHSA) experience hope? What inspires hope, according to persons experiencing MHSA problems?	Cooperative action research approach with individual semi-structured interviews	Nine persons with MHSA problems	Daring to believe that something better is possible. You need something to hold on to when you are looking for the light at the end of the tunnel. You need some people you can trust and who have faith in you. You have to decide whether you want to go on or not.
[55]	What are the personal narratives of recovery of persons with substance abuse problems?	Phenomenological narratives—written narratives	Fourteen persons with MHSA	Recovery as a long process and involving changes in significant aspects of the persons’ lives for the better: Different prerequisites for the recovery processes Improvement as: “Improvement is the distance between who I felt I was and who I feel I am.” Building capacity for change taking a long time, requiring patience Requires continuous work with oneself Recovery is a natural part of life Recovery in terms of meaningful everyday life Focus on resources and future Involves re-establishing social life and social relations
[56]	To explore how young adults with co-occurring MHSA problems experience a sense of belonging in their local environment, and facilitators and barriers related to belonging	Hermeneutic-phenomenological approach with in-depth interviews	Seven young adult users	Can’t find anything to relate to in the mainstream Balancing between mainstream and outsider life Trying to get a stronger foothold in the mainstream
[57]	Explore and describe recovery as experienced by young adults who live with co-occurring MHSA	Qualitative, individual interviews	Seven young adult service users of municipal community MHSA services	The person is more than the diagnosis Users and professionals create different identities Focusing on possibilities and resources
[58]	Explore therapists’ views of the processes of recovery in bipolar disorders	A reflexive, collaborative approach with semi-structured individual interviews	Twelve professional providers	A “puzzling given” (as a fact that is incomprehensible) related to the complexity, unpredictability, and irregular patterning of bipolar disorders, pointing to recovery as complex Users as the protagonists of the healing process—personal qualities and strength, being resilient, and developing personal strategies to deal with problems The heroic fighter does not always win—dealing with disappointments and fights lost; respecting users’ hard work when unsuccessful
[59]	Explore and describe recovery as experienced by persons living with co-occurring MHSA	Phenomenological individual interviews	Eight persons with recovery experiences	Four dimensions of recovery: feeling useful and accepted coming to love oneself mastering life emerging as a person. Insecure and inadequate housing and limited solutions to financial problems as major obstacles to recovery.
[60]	To explore and describe service users’ experiences with peer support relationships, support and collaboration.	Hermeneutic-phenomenological approach with focus group interviews	Twenty-six service users with MHSA problems	Relationships and collaboration with peer supporter workers as positive. Challenges in peer support relationships and collaboration in terms of creating hope, equality, trust, and freedom to be helpful in other ways than those employed by professionals
[61]	How do relatives of people with mental illness describe their experiences of hope?	Phenomenological, descriptive approach with focus group interviews	Fifteen relatives of people with mental illness	Basic hope as a basic attitude, as a fundamental resource in life and a universal human condition of life, in line with love. Everyday hope as hoping for a little more improvement and as qualitatively “small” hopes; linked to processing guilt, related to environmental factors, and experiencing hope in relation to one’s family members’ life situation.
[62]	Stories of hope and recovery in MHSA	Written narratives	Two men with experience of MHSA	Stories providing images of the self and a way of sharing oneself Stories as ways to move forward to opportunities for change and hope. Stories carrying contradictions Stories manifested through telling and clarifying oneself Stories of hopelessness as the beginning of hopefulness Stories as sharing
[63]	To examine the role of social relationships in reaching and maintaining stable recovery after many years of substance use disorders	Individual interviews, narrative analysis	Eighteen adult service users with at least five years of stable recovery	Putting things straight with oneself and those around one Becoming responsible through boundary-setting practices Experiencing a strong sense of duty
[64]	What do young adults with co-occurring MHSA find challenging in relation to belonging in their local communities?	In-depth individual interviews	Seven young adult users of municipal MHSA services	The need to accept one’s life and its surrounding structures: accepting one’s life story, and accepting the rules Being caught between conflicting social worlds—a choice between belonging to outsider life or the mainstream Moral fumbling in choices and actions—unprepared to be full participants in the mainstream and faltering moral and emotional connections to the mainstream along a continuum of condemnation versus self-blame
[65]	To explore and describe first-person experiences of relational recovery in persons with MHSA conditions	In-depth individual interviews	Eight adult service users with MHSA problems at various stages of recovery	Social relationships viewed as both supportive and hindering recovery: Choosing one’s child (parenting as the motivation for recovery) Living with loneliness and a painful past Sacrificing everything for one’s partner Regaining trust and support
[66]	How do persons with co-occurring MHSA problems in supported housing experience belonging? How do residential support staff experience promoting a sense of belonging for this group?	Collaborative and reflexive individual interview ing and focus group interview	Residents of a supported housing facility and the staff	The experience of belonging in relation to the contribution of the community and contextual factors in supported housing, such as: I do not go to sleep in my pajamas (supported housing being a house rather than a home and a lack of sense of belonging) Do I have a choice? (Experiences of belonging connected to choice and having resources to make decisions on one’s behalf) Be kind to each other (the meaning of living with others)
[67]	To explore embodying experiences of nature related to recovery in everyday life for persons with eating disorders	Hermeneutic-phenomenological approach with individual interviews	Eight persons with experience of eating disorders	Experiences of nature as accentuating feelings of calmness and an engagement of the senses. Nature experienced as a non-judgmental environment that also provided room for self-care. Meeting nature through one’s body, particularly one’s feet, facilitating contact with the body and challenging the body-mind dichotomy

## Data Availability

All the included studies are in Table 1.
